# Ex situ phytoremediation trial of Sardinian mine waste using a pioneer plant species

**DOI:** 10.1007/s11356-021-14710-y

**Published:** 2021-06-18

**Authors:** Maria Enrica Boi, Giovanna Cappai, Giovanni De Giudici, Daniela Medas, Martina Piredda, Marco Porceddu, Gianluigi Bacchetta

**Affiliations:** 1 Department of Chemical and Geological Sciences, University of Cagliari, Cittadella Universitaria di Monserrato, S.S. 554 bivio per Sestu (CA), 09042 Monserrato, Italy; 2 Department of Civil and Environmental Engineering and Architecture, University of Cagliari, Piazza d’Armi 1, 09123 Cagliari, Italy; 3 Department of Life and Environmental Sciences, Centre for Biodiversity Conservation (CCB), University of Cagliari, Viale Sant’Ignazio da Laconi 11-13, 09123 Cagliari, Italy; 4 Sardinian Germplasm Bank (BG-SAR), Hortus Botanicus Karalitanus (HBK), University of Cagliari, Viale Sant’Ignazio da Laconi, 9-11, 09123 Cagliari, Italy

**Keywords:** Asteraceae, Metal tolerance, Mine wastes, Phytostabilization, Pollutant metals, Mediterranean vascular flora

## Abstract

The mitigation of metals contamination is currently a crucial issue for the reclamation of mine sites. Indeed, mine wastes are often disposed in open dumps and consequently pollutants are subjected to dispersion in the surrounding areas. In this study, the potential use of *Helichrysum microphyllum* subsp. *tyrrhenicum* for phytostabilization was evaluated in ex situ conditions. Ninety specimens were randomly selected and were planted in three substrates (reference substrate, mine waste materials, and mine wastes with compost). Mineralogical compositions of substrates, rhizosphere, and roots were assessed through X-ray diffraction (XRD). Zn, Pb, and Cd concentrations of substrates, rhizosphere, soil pore waters, and plant tissues were determined. The phytostabilization potential was determined through the application of biological accumulation coefficient (BAC), biological concentration factor (BCF), and translocation factor (TF). Moreover, survival and biometric parameters were assessed on plant specimens. The polluted substrates and related rhizosphere materials were mainly composed of dolomite, quartz, pyrite, and phyllosilicate. Zn was the most abundant metal in substrates, rhizosphere, and soil pore waters. XRD analysis on roots showed the presence of amorphous cellulose and quartz and Zn was the most abundant metal in plant tissues. *H. microphyllum* subsp. *tyrrhenicum* restricts the accumulation of the metals into roots limiting their translocation in aereal parts, indicating its potential use as phytostabilizer (BCF, BAC, TF < 1). Survival and growth data showed a great adaptability to different substrates, with an evident positive effect of the implementation of compost which increased the plant survival and decreased the metals uptake into roots.

**Supplementary Information:**

The online version contains supplementary material available at 10.1007/s11356-021-14710-y.

## Introduction

Mine waste dumps are limiting environments for the growth and establishment of most plant species. These unfavorable conditions are related to the properties of mine substrates such as: the absence of a top soil, the lack of nutrients (K, N, and P) and organic matter, the poorly developed structure, and the high concentration of toxic elements like heavy metals (Mendez and Maier 2008; Baker et al. 2010; Anawar et al. 2011; De Agostini et al. 2020). Mining activity and in prior way its waste materials are some of the main sources of toxic metal pollution in biogeochemical spheres. Hence, the mitigation of contaminant dispersion in mine sites is currently an environmental and sanitary challenge. Indeed, mine wastes are often disposed in open dumps mainly made of fine particles, which can be subjected to dispersion by wind and water erosion, especially in arid and semiarid environments (Munshower 1994; Mendez and Maier 2008; Sims et al. 2013; Doumas et al. 2018; Zine et al. 2021). Last but not the least, mining activities can seriously alter ecosystems in terms of soil structure and related functions with loss of biodiversity and land degradation (Ahirwal and Pandey 2021).

Despite these unfavorable conditions for plant growth, several species have developed different adaptations to survive in these environments, like metal-tolerance capability. Plants who are sufficiently resilient to colonize metals’ polluted areas and have developed metal tolerance can have an unquestionable role in the development of a long-term plant canopy (Freitas et al. 2009). Taking advantage of these benefits, plants can give their contribution in the reclamation of these sites by applying phytoremediation, that is a low impact and cost-effective strategy and for the recovery of mine areas (Freitas et al. 2009; Favas et al. 2014; Bacchetta et al. 2015). In order to achieve this purpose, it is necessary to select the best performing plant species with a special focus on native taxa. Owing this issue, several reasons promote the implementation of autochthonous plants over non-native species: (i) they are adapted to local climate/adverse conditions (Pandey and Singh 2012; El Hasnaoui et al. 2020) and are stronger in terms of growth, survival, and reproduction under environmental stress (Midhat et al. 2017); (ii) their use can preserve the plant diversity of the natural territories (Cao et al. 2009; Bacchetta et al. 2012, 2015; Concas et al. 2015a); (iii) they help to start the rehabilitation of vegetational dynamics by the improvement of the physical-chemical properties of the substrates (Vacca 2000; Vacca et al. 2017), and (iv) they help to the establishment of a long-term plant canopy on mine wastes with a relatively low cost inputs and limited maintenance (Bacchetta et al. 2007a; Pandey et al. 2015; Monaci et al. 2020; Zine et al. 2020). All these points are pieces of a more holistic and sustainable approach in phytoremediation (Pandey et al. 2015; Pandey and Omesh Bajpai 2019).

An important role in phytoremediation can be played by soil amendments since they can improve the plant growth modifying the physical-chemical properties of mine substrates and the bioavailability of metals (Song and Greenway 2004; Bacchetta et al. 2012, 2015). Among the available soil amendments, compost can optimize phytoremediation by lowering the metal availability in substrates and the plant’s tissue uptake and increasing the content of nutrients and organic matter in substrates (Bacchetta et al. 2015). Moreover, compost modifies the structure of substrate through the improvement of the bulk’s water retention, density, and ventilation (Fagnano et al. 2011). The result of this interaction leads to a better roots penetration and consequently results in a better feeding of the plant.

Sardinia (Italy) had an important mining history, which left a burdensome environmental legacy: after the shutdown of mines, few containment activities of mine tailing dumps and mitigation of the related metals impact were developed (Jiménez et al. 2014; Bacchetta et al. 2018), leaving consequently huge quantities (approximately 70 million of m^3^) of polluted materials subjected to erosion and dispersion (RAS – Regione Autonoma della Sardegna 2003; Bacchetta et al. 2018; Boi et al. 2020a, b). Along the last 15 years, several studies were carried out on the Sardinian mine context through field sampling, in situ and ex situ phytoremediation experiments. In details, these studies highlighted that different autochthonous species are able to grow in these environments and to tolerate extremely high heavy metal concentrations (e.g., Cao et al. 2004, 2009; Jiménez et al. 2005, 2011, 2014; Bacchetta et al. 2012, 2015, 2017, 2018; Concas et al. 2015a; De Giudici et al. 2015, 2017; Medas et al. 2015, 2019; Fancello et al. 2019; Boi et al. 2020a; De Agostini et al. 2020).

These abandoned mining areas are anyway rich in terms of plant diversity (Angius et al. 2011; Angiolini et al. 2005; Bacchetta et al. 2007a, b, c; Zavattero et al. 2005, 2006) and in particular of endemic taxa: for example, in the Monteponi mine area (Iglesias, South West Sardinia), endemic species are 18.1% of the local flora (Zavattero et al. 2005). Indeed, several endemic and/or threatened plant species like *Echium anchusoides* Bacch., Brullo & Selvi, *Brassica insularis* Moris, *Clinopodium sandalioticum* (Bacch. & Brullo) Bacch. & Brullo ex Peruzzi & F.Conti, *Staphisagria requienii* (DC.) Spach subsp. *picta* (Willd.) Peruzzi, *Euphorbia pithyusa* L. subsp. *cupanii* (Guss. ex Bertol.) Radcl.-Sm., *Helichrysum microphyllum* Cambess. subsp. *tyrrhenicum* Bacch., Brullo & Giusso, *Iberis integerrima* Moris, *Linum muelleri* L., *Limonium merxmulleri* Erben, *Ptilostemon casabonae* (L.) Greuter, *Santolina insularis* (Gennari ex Fiori) Arrigoni, and *Scrophularia canina* subsp. *bicolor* (Sibth. & Sm.) Greuter are recognizable on these sites.

For the aim of this study, a lab-scale phytoremediation test was carried out on contaminated matrices from the Sulcis–Iglesiente area (South West Sardinia, Italy) using *H. microphyllum* subsp. *tyrrhenicum* (hereafter *H. tyrrhenicum*), which is an endemic plant species of Sardinia and Corsica (Bacchetta et al. 2003). Previous studies concerning this taxon highlighted its great adaptability to restrictive environments as pioneer of mine wastes (Angiolini et al. 2005; Bacchetta et al. 2009), the metal tolerance towards Zn, Pb, and Cd (Cao et al. 2004; Bacchetta et al. 2017, 2018; Medas et al. 2018; Boi et al. 2020a), and the capability of its seeds to germinate and seedlings to survive under very high concentrations of Zn and Pb (Boi et al. 2020b).

The aims of this experimental study were to evaluate: (i) the capability of *H. tyrrhenicum* to tolerate high concentration of Zn, Pb, and Cd, assessed in terms of plant survival and growth; (ii) its phytoremediation potential towards these metals by assessing the biological indexes regarding metal accumulation in plant tissue, and (iii) the effectiveness of organic amendments (compost) in the mitigation of heavy metal stress.

## Materials and methods

### Study area

**Fig. 1:**
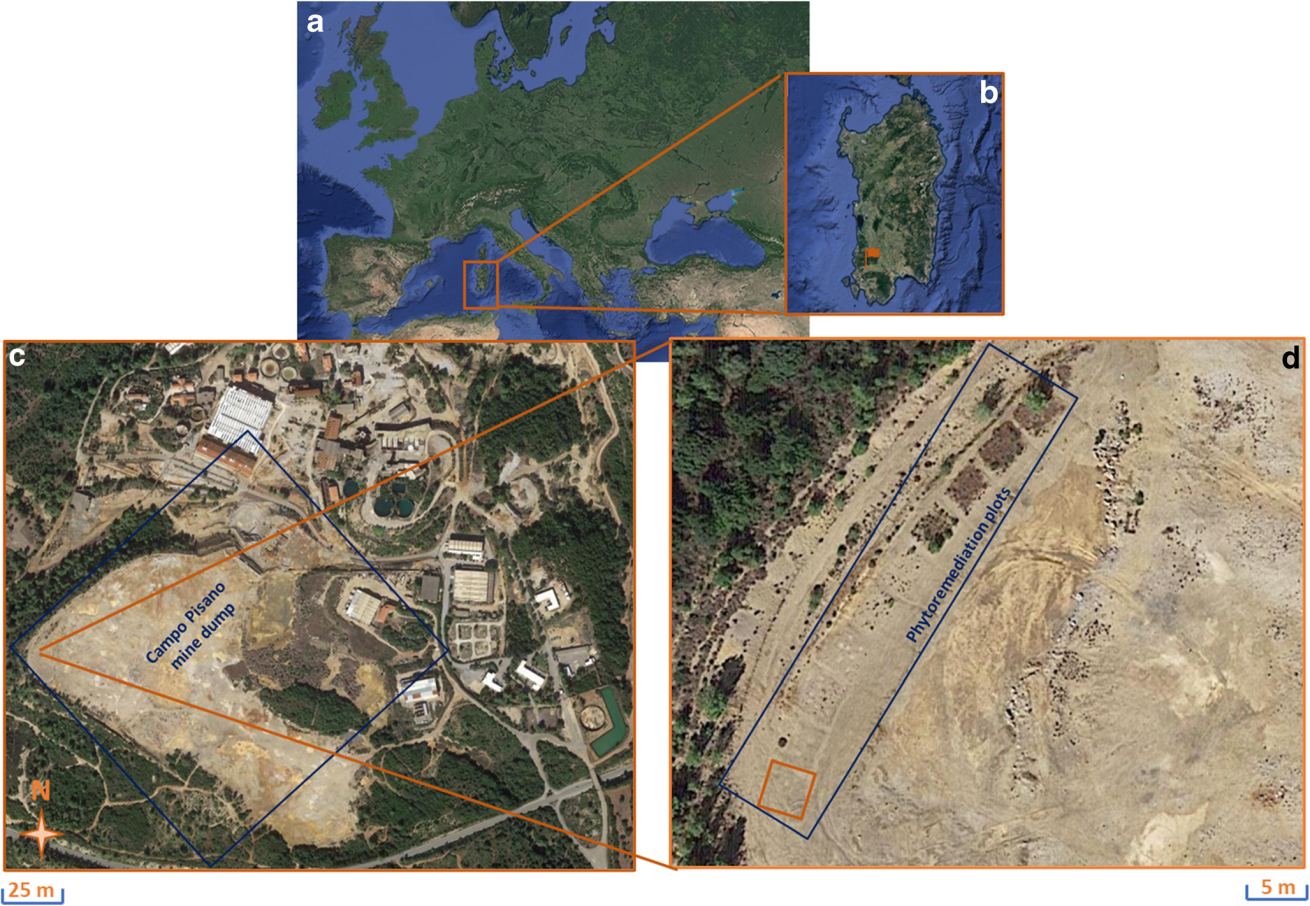
**a** Position of Sardinia (Italy); **b** localization of Campo Pisano mine dump in the southwest of Sardinia (orange flag); **c** the Campo Pisano mine dump (blue square); **d** sampling point inside the plot (orange square) of a previous phytoremediation experiment (blue rectangle)

The area of interest (Fig. 1a–d) is Campo Pisano mine dump (Iglesias, South West Sardinia, Italy), and it is characterized by a thermo-mediterranean bioclimate (Rivas-Martínez et al. 2002; Bacchetta et al. 2009). The mine wastes derived of grounding and flotation processes were settled during the period of activity. These materials are characterized by a high concentration of metals (especially Zn, Pb, and Cd) and poor agronomic properties, i.e., lack of nutrients and organic matter (Bacchetta et al. 2012, 2015, 2018). Nearby the area, the fine materials have been found dispersed due to the aeolian and water dispersion, impacting also living organisms (Cidu et al. 2001, 2005; Varrica et al. 2014; Concas et al. 2015a; Bacchetta et al. 2015, 2018; De Giudici et al. 2017; Boi et al. 2020a). Moreover, the mine wastes are highly heterogeneous in terms of metal concentrations (Boni et al. 1999; Bacchetta et al. 2018) mainly due to the different extraction methods used along the time in which the mine exploitation was effective.

### Experimental design

Ninety-five specimens of *H. tyrrhenicum* (approximately 2 years of age) were randomly selected for this experiment from a largest set. Plants specimens were provided by “Forestas Agency” of “Regione Autonoma della Sardegna” and produced at the nursery of Campu S’Isca (Villacidro, SW Sardinia). These specimens were grown in a substrate without heavy metals contamination before this experiment and consequently, they had to adapt to these new stressing conditions. Five specimens were used for a preliminary characterizations of metal content and plant size (see the following paragraphs for more details). The remaining ninety specimens were randomly divided in three different groups and planted in three different substrates (thirty specimens each): (i) unpolluted reference substrate (RS; Control treatment); (ii) Campo Pisano (CP) mine waste collected within an experimental plot (Fig. 1d, orange square) of a previous phytoremediation experiment reported in Bacchetta et al. (2012) (treatment with mine substrate), and (iii) Campo Pisano mine waste, collected from the same plot, amended with 10% w/w of compost (CPC; treatment with mine substrate amended with compost). The choice of this compost percentage was made taking into account a previous ex-situ experimental study on Mediterranean native plants, in which amending mine substrate with this percentage showed its effectiveness in terms of plant survival and growth (Bacchetta et al. 2015). Compost was provided by “Tecnocasic” (Cagliari, Italy) and derived from the aerobic stabilization of selected municipal solid waste organic fractions.

**Fig. 2:**
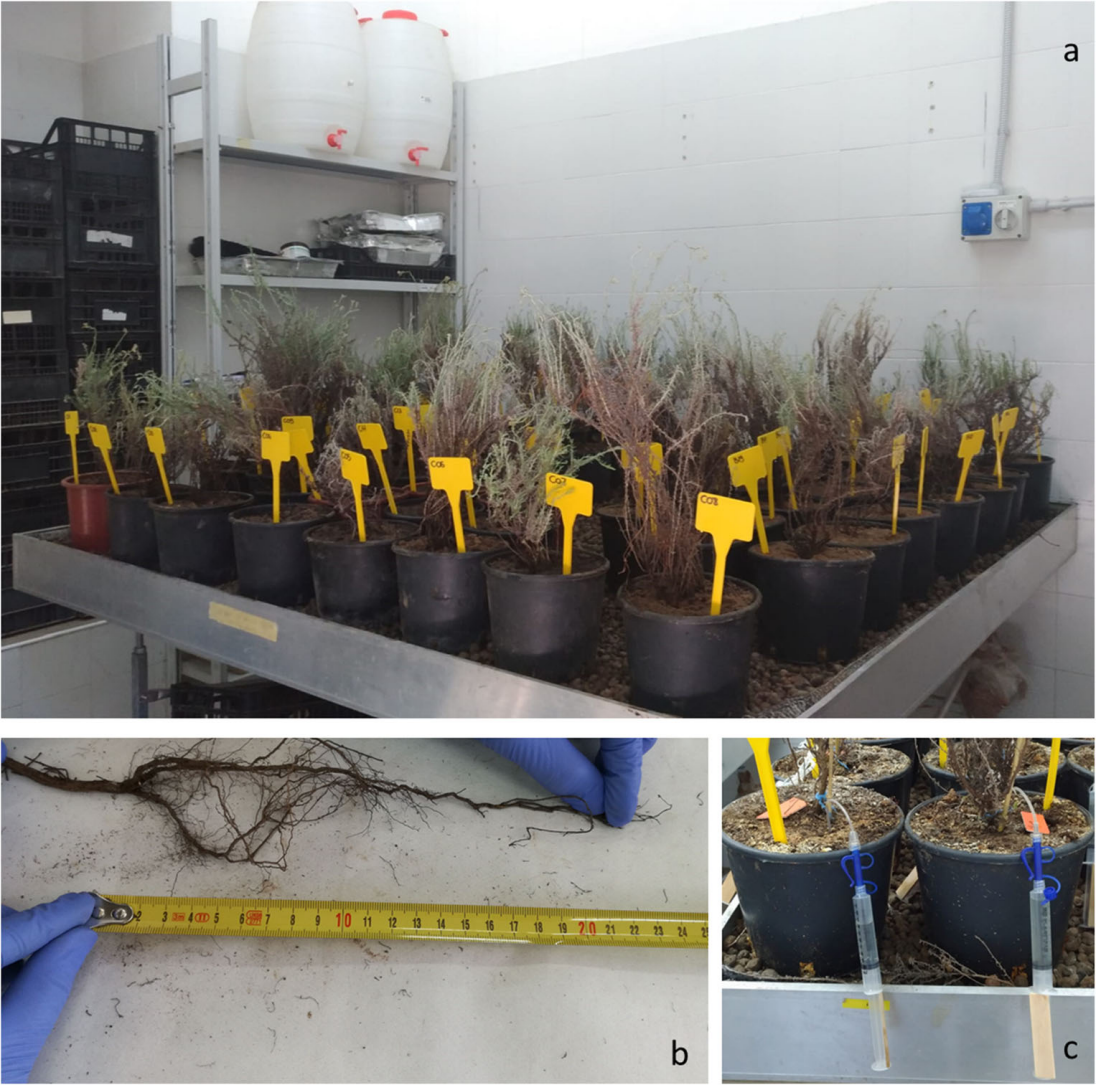
Some phases of the lab-scale experiment: **a** the trial at greenhouse condition; **b** measurent of biometric parameters; **c** sampling of soil pore water

The Campo Pisano mine waste (aproximalely 200 kg) was randomly collected using a shovel along the selected plot at a maximun deep of 30 cm, divided and stored in 5 kg plastic bag for transport in the laboratory where each substrate was separately created: approximately 90 kg of the mine waste (CP) was mixed several times with a shovel in order to homogenize it; the other 90 kg of mine waste were additionated with the compost and mixed several time with the shovel, for obtaining the amended substrate (CPC); the reference substrate (RS) was created ad hoc using 35% of a commercial soil, 30% of sand, 30% of gravel, and 5% of peat. Specimens of *H. tyrrhenicum* were individually planted into 3 L polyethylene pots, which were filled with one of the specific substrate (Fig. 2a). The trial was performed at greenhouse conditions for 6 months with a constant temperature at 20 °C, humidity at 65–70%, and photoperiod of 12 h of light and 12 h of dark (Fig. 2a). Specimens were watered twice a week with 75 mL of distilled water. These conditions were selected based on a previous phytoremediation laboratory experiment carried out on Mediterranean vascular plant species (Bacchetta et al. 2015).

Before planting (T0), the concentration of Zn, Pb, and Cd in the substrates and the length of the roots and stems, diameter of the stems, weight of roots, and epigean organs were measured (Fig. 2b) on five specimens randomly selected among the total number of ninety-fives collected for the experimental campaign. Successively, these parameters were assessed on five pot and related specimens of each treatment randomly selected and sacrificed at three different times during the experiment: T1, after 1 month; T2, after 3 months; T3, after 6 months. In addition, metal concentrations in rhizosphere materials were evaluated at T1, T2, and T3. Rhizosphere is defined as the narrow portion of soil which behaves as interface soil, plant roots, water, microbes, and air (Boi et al. 2020a). Plant survival was weekly assessed and soil pore waters were monthly collected (six times during the experiment) in five random pots (chosen at the beginning of the trial) through rhizon sampler (Fig. 2c), as proposed by other works (Di Bonito et al. 2008; Concas et al. 2015b). Rhizometer is a kind of tension sampler made by a little plastic tube wrapped of ceramic material. It is used by inserting the tube near the roots catching zone. After, it is possible to extract the soil pore water connecting a syringe and creating the necessary vacuum to have suction. Moreover, the mineralogical analysis in terms of mineral composition of rhizosphere materials and roots was carried out at each sampling time.

### Substrates, rhizosphere materials, and soil pore water analysis

After plant harvesting, substrates were divided from roots and the content of the five pots was mixed, twice quartered and sieved at < 2 mm. Rhizosphere materials were obtained shaking roots of each plant specimen into a plastic bag and after wiping them with a brush.

Mineralogical compositions of substrates and rhizosphere materials were assessed on samples ground in agate mortar and then obtained through X-ray diffraction (XRD) using a θ–2θ diffractometer (PANalytical X’PERT MPD) with Cu Kα radiation (1.5418 Å). Peaks for the mineral were attributed using “X’Pert Highscore plus” software and in accordance to the Powder Diffraction Cards.

Total content and bioavailable fraction of Zn, Pb, and Cd of substrates were assessed in a composite sample of five pots. The metal concentration in rhizosphere materials was measured for each plant specimen. The total metal content in substrates and rhizosphere materials was assessed on duplicate samples through microwave-assisted acid digestion (Start D milestone), following the procedure described in Bacchetta et al. (2018) and Boi et al. (2020a).

The bioavailable metal concentration was evaluated through a single extraction on duplicate samples of substrates, using a solution of diethylenetriaminepentacetic acid (DTPA), calcium chloride (CaCl_2_), and triethanolammine (TEA) buffered at pH 7.3, according to the procedure for not acidic soils proposed by Linsday and Norvell 1978) and reported by the Italian law (GURI 1999).

All the obtained solutions of substrates, rhizosphere, and bioavailable fraction were filtered at 3–5 μm and analyzed by Inductively Coupled Plasma Emission Spectrometry (ICP-OES, Perkin Elmer Optima DV 7000) using the following wavelengths: Zn 213.857 nm, Pb 220.353 nm, and Cd 228.802 nm (Bacchetta et al. 2018; Boi et al. 2020a). Measurements of pH of substrates were performed following the Italian official methods (GURI 1999).

Soil pore waters were collected and stored in plastic sample holder and immediately stabilized with 1% of HNO_3_ and then, the investigated metals were measured by ICP-OES at the same wavelengths used for substrates and rhizospheres.

### Plant analysis

Specimens were divided into roots and epigean organs and subsequently, the length of roots and epigean organs and the diameter of stem were measured. Plants were washed with tap water and with deionized one for several times (> 6), in order to remove soil particles, which may remain stuck on the surface of the plant. Finally, roots and epigean organs were dried at 40 °C in an oven (Binder GmbH) until they reached a constant weight. Roots and epigean organs were ground (< 40 μm) with an electric grinder (Ultra Centrifugal MillZM200, Retsch GmbH). For the mineralogical analysis, the obtained roots powder were ground once again in an agate mortar and investigated by XRD, following the same methodology used for substrates and rhizosphere materials. It is a well-known fact that different kind of biominerals occurs in plant tissues, performing several roles like protection towards herbivory and pathogen, alleviation from water and temperature stress, detoxification, and accumulation systems of toxic elements (He et al. 2014). Biomineralization was observed in *H. tyrrhenicum* specimens spontaneously growing on mine waste (Bacchetta et al. 2018; Boi et al. 2020a). However, in the frame of this ex-situ experiment, the mineralogical analysis was carried out in order to verify the presence of biominerals also on specimens which have to adapt to metals stressing conditions.

Zn, Pb, and Cd concentrations were evaluated on duplicated samples by a microwave-assisted acid digestion following the procedure described in Boi et al. (2020a). The metal concentration in filtered solution (3–5 μm) was analyzed by ICP-OES using the same wavelengths used for substrates, rhizosphere, and soil pore waters.

### Quality assurance and quality control

Different reference materials and blank solutions were used in order to ensure the reliability of the analytical methods in the analysis of metal content of substrates, rhizospheres, and plant tissues. In details, for substrates and rhizospheres, the “GSS-4, limy-yellow soil” was used as reference material, whereas for plant samples, “GSV-2 bush twigs and leaves” and “INCT-PVLT-6 Polish Virginia Tobacco leaves” were used. Reference materials and blank solutions were processed using the same acid digestion procedure used for the related samples. Morever, during the ICP-OES analysis, the “EnviroMAT-DrinkingWater High” (EP-H-3, SCP Science, ref.140-025-032) reference solution was used in order to verify the precision and accuracy of the instrument.

### Assessment of the phytoremediation potential

Different biological indexes were calculated in order to evaluate the behavior of *H. tyrrhenicum* towards Zn, Pb, and Cd:

1. Biological accumulation coefficient (BAC, Brooks 1998)

1 $$ \mathrm{BAC}=\frac{\left[ Hm\  eo\right]}{\left[ Hm\ s\right]} $$

where [*Hm eo*] is the heavy metal concentration in epigean organs and [*Hm s*] is the heavy metal concentration in substrate. It is used to estimate the accumulation of metals in areal organs with respect of the content in the substrate.

(2) Biological concentration factor (BCF, Yoon et al. 2006)

2 $$ \mathrm{BCF}=\frac{\left[ Hm\ r\right]}{\left[ Hm\ s\right]} $$

where [*Hm r*] is the heavy metal concentration in roots and [*Hm s*] is the heavy metal concentration in the substrate. It gives information about the catching ability of roots towards substrate metal content.

(3) Translocation factor (TF, Brooks 1998)

3 $$ \mathrm{TF}=\frac{\left[ Hm\  eo\right]}{\left[ Hm\ r\right]} $$

where [*Hm eo*] is the heavy metal concentration in epigean organs and [*Hm r*] is the heavy metal concentration in roots. It is used in order to evaluate the metal translocation from roots to epigean organs.

BAC and BCF were both calculated also considering the bioavailable fraction of the metals (BAC *bf* and BCF *bf*) since only a small fraction of the total content is actually available for roots (Adamo et al. 2013).

### Statistical analysis

One-way ANOVA was used to evaluate the differences between substrates and to highlight possible time variations (from T1 to T3) in terms of concentration of Zn, Pb, and Cd in the plant tissues (roots and epigean organs), rhizosphere materials, as well as for each biological index (BCF, BCF *bf*, BAC, BAC *bf*, and TF). Furthermore, one-way ANOVA was applied on the assessed biometric parameters and soil pore waters. Significant differences highlighted by ANOVA were then analyzed by a post-hoc pairwise comparisons t-test (Student-Newman-Keuls). All statistical analyses were performed using R v. 3.0.3 (R Development Core Team 2014).

## Results and discussion

### Chemical and mineralogical characterization of the substrates and rhizosphere materials

**Fig. 3:**
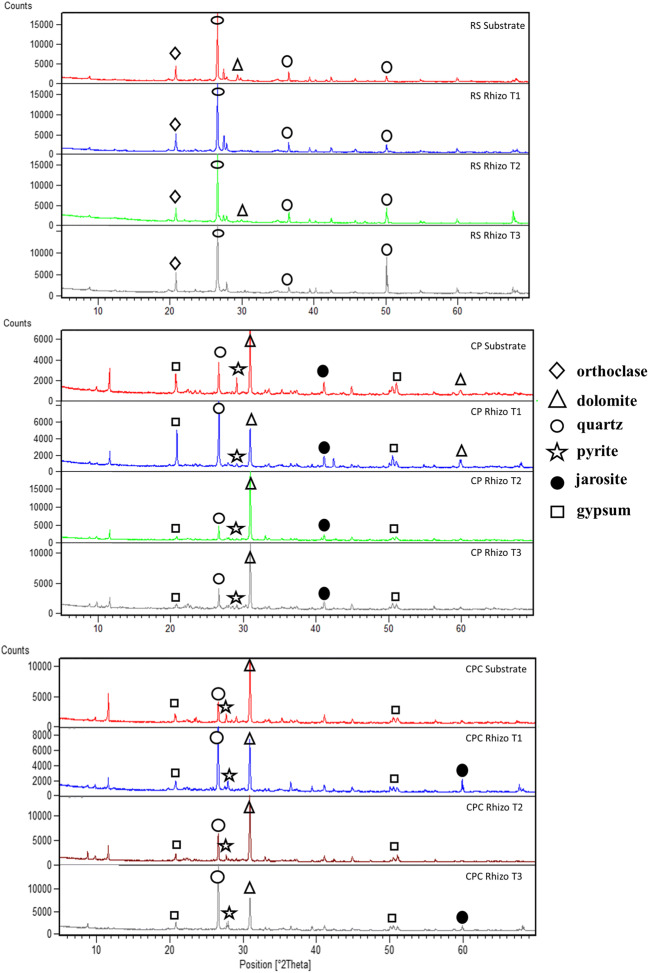
Comparison of XRD spectra of selected sample of the substrates and rhizosphere materials in RS (**a**), CP (**b**), and CPC (**c**) among sampling times; rhizo, rhizosphere materials; T1, after 1 month; T2, after 3 months; T3, after 6 months

The mineralogical compositions of the substrates and rhizosphere materials collected during the experiment are reported in Table 1. RS substrate and rhizosphere materials were mainly composed of quartz (SiO_2_), phyllosilicates, and feldspars (albite, anorthite, orthoclase, microcline, and sanidine) and no minerals linked to polluting metals, such as pyrite (FeS_2_), goethite (Fe^+3^O(OH)), jarosite (KFe^3+^_3_(SO_4_)_2_(OH)_6_), and anglesite (PbSO_4_), were observed. The polluted substrates (CP and CPC) and related rhizosphere materials were mainly composed of dolomite (CaMg(CO_3_)_2_), quartz, pyrite and phyllosilicate, and some secondary minerals like goetithe, gypsum (CaSO_4_·2(H_2_O)), jarosite, and anglesite, according to previous mineralogical characterizations on samples of Campo Pisano mine dump (De Giudici et al. 2015; Bacchetta et al. 2017, 2018; Boi et al. 2020a). In details, quartz and dolomite form the gangue minerals of the ore body and are often recognized together with barite (BaSO_4_) and Fe-oxy-hidroxydes (De Giudici et al. 2015), whereas pyrite is associated with sulfide deposits in SW Sardinia (Boni et al. 1999, 2003) and gypsum and jarosite derive from the dissolution of Ca-carbonates and the oxidation of pyrite, respectively. It is interesting to note that mineral composition of rhizosphere materials did not change during all the trial (Table 1 and Fig. 3). Probably, a long-term in situ phytoremediation experiment could be necessary to highlight an evolution in rhizosphere mineralogy.

**Table 1:** Mineralogical composition of substrates and rhizosphere in RS, CP, and CPC. The black points indicate the presence of the mineral; S, substrate; Rz, rhizosphere materials; T0, before planting; T1, after 1 month; T2, after 3 months; T3, after 6 months

		Time	Dolomite	Quartz	Phyllosilicates	Albite	Anorthite	Orthoclase	Sanidine	Microcline	Pyrite	Goethite	Gypsum	Jarosite	Anglesite
RS	S	T0	●	●	●	●	●	●	●						
	Rz	T1		●	●	●	●	●	●						
	Rz	T2	●	●	●	●		●		●					
	Rz	T3		●	●	●	●	●							
CP	S	T0	●	●							●		●	●	
	Rz	T1	●	●	●	●					●	●	●	●	
	Rz	T2	●	●	●						●		●		●
	Rz	T3	●	●	●	●				●	●		●		●
CPC	S	T0	●	●							●		●		
	Rz	T1	●	●	●	●		●		●	●		●		
	Rz	T2	●	●	●	●		●		●	●	●	●		
	Rz	T3	●	●	●	●		●		●	●	●	●

The chemical characterization (Zn, Pb, Cd concentration, and pH values) of the substrates used during the experiment (from T0 to T3) is reported in Table 2. All the matrices were slightly alkaline (pH 7.60–7.68) and the assessed pH values of CP and CPC are consistent with carbonates lithology of Campo Pisano area (Bechstädt and Boni 1994; Bacchetta et al. 2015, 2018). The mine waste (CP) showed the highest values for the three metals, whereas the reference substrate (RS) showed the lowest ones. Metal content in CPC was always lower than in CP, indicating that compost addition to Campo Pisano’s mine waste has produced a diluting effect. Indeed, the concentration of metals in compost was 154 ± 6 mg kg^−1^, 38.38 ± 0.01 mg kg^−1^, and < DL for Zn, Pb, and Cd, respectively, markedly lower than that in CP. Moreover, the assessed values of metal content in compost were consistent with those assessed in Bacchetta et al. (2015), though it was provided by different factories. The most abundant metal in each matrix was Zn, followed by Pb and Cd. However, quite variable values in metal concentration were assessed in CP and CPC, reasonably due to the heterogeneity of the mine waste (Boni et al. 1999), as also reported by other authors (Cao et al. 2009; Bacchetta et al. 2015, 2018) but they also indicate that the homogenization of the substrate prior to planting was not completely effective.

**Table 2:** Chemical characterization of substrates during the experiment (mean ± SD, n = 2); RS, reference substrate; CP, Campo Pisano; CPC, Campo Pisano + compost; tot, total concentration; *bf*, bioavailable fraction; T0, before planting; T1, after 1 month; T2, after 3 months; T3, after 6 months

			Matrix		
			RS	CP	CPC
Zn	tot (mg kg^−1^)	T0	152 ± 14	11,442 ± 1018	6726 ± 164
		T1	318 ± 10	9227 ± 359	6052 ± 489
		T2	231 ± 14	8123 ± 790	7610 ± 215
		T3	152 ± 30	7338 ± 496	7874 ± 5
	*bf* (mg kg^−1^)	T0	6.21 ± 0.50	45 ± 0.91	135 ± 10
	*bf* (%)	T0	4.08	0.39	2
Pb	tot (mg kg^−1^)	T0	82 ± 13	2478 ± 112	612 ± 96
		T1	133 ± 2	1815 ± 188	1046 ±153
		T2	102 ± 7	1741 ± 275	1575 ± 210
		T3	84 ± 20	1482 ± 153	1204 ± 40
	*bf* (mg kg^−1^)	T0	2.95 ± 0.16	9.24 ± 0.18	22.24 ± 1.60
	*bf* (%)	T0	3.52	0.37	3.63
Cd	tot (mg kg^−1^)	T0	0.48 ± 0.13	65 ± 15	34 ± 5
		T1	2.60 ± 0.06	49 ± 4	26 ± 3
		T2	1.94 ± 0.01	39 ± 1	36 ± 2
		T3	1.17 ± 0.01	47 ± 2	52 ± 35
	*bf* (mg kg^−1^)	T0	0.05 ± 0.01	0.55 ± 0.02	2.29 ± 0.13
	*bf* (%)	T0	10.4	0.84	6.73
pH		T0	7.65	7.60	7.68

The high metal content assessed in CP and CPC confirmed the extreme metal pollution of this area (Bacchetta et al. 2012, 2015, 2018; Concas et al. 2015a; Lai et al. 2015; Boi et al. 2020a). Bacchetta et al. (2015) have also shown that this substrate is characterized by a scarce presence of organic matter as organic carbon (C _org_ % = 1.8) and nutrient as nitrogen and phosphorous (N % = 0; P = 360 mg kg^−1^). Hence, carbon was mainly present in inorganic forms as calcite (48.0 ± 4.4 g kg^−1^) and dolomite (430.0 ± 10.1 g kg^−1^), consistently also with mineralogical characterization. As far as the matrix texture is concerned, the above mentioned work reported that it consists of fine-grained particles (< 425 μm, 86.2%), accounting the 27% of lime (< 50 μm) and 3% of silt (< 2 μm) with deleterious consequences for the potential growth and penetration of roots caused by substrate self-compaction.

It is noteworthy that metal concentrations assessed in CP and CPC are far greater than the threshold contamination levels set by Italian law (Decreto Legislativo n.152 2006) for industrial use of soil (1500, 1000, 15 mg kg^−1^ for Zn, Pb, and Cd, respectively), with the exception of the Pb in CPC, which was below its threshold. The compost used for this study showed a Zn, Pb, and Cd concentration within the limits established by the European Union (Zn: 2500–4000 mg kg^−1^; Pb: 750–1200 mg kg^−1^; European Communities Council Directive 1986) and by the Italian law (Zn ≤ 500 mg kg^−1^; Pb ≤ 140 mg kg^−1^; Decreto legislativo n. 217 2006) for agricultural use.

Despite the high total content observed in polluted matrices, very low concentrations of bioavailable metals (*bf* in Table 2) were observed in all substrates (i.e., < 1% in CP) showing that only a little amount is available for plant’s roots. In CPC, a bioavailability higher than in CP tests was measured, despite several studies reported the ability of compost to reduce the bioavailability of metals (Bacchetta et al. 2012, 2015). However, its effect on metal bioavailability can be contradictory (Baldantoni et al. 2010; Beesley and Dickinson 2010; Fagnano et al. 2011) and this ambiguity can depend on different factors, such as the origin of organic material, kind of soil, involved metals, and the formation of soluble metal-organic complexes which can increase or decrease the bioavailability (Baldantoni et al. 2010; Fagnano et al. 2011; Hattab et al. 2014). The increase in metal bioavailability can be ascribable to the formation of metal chelates from fragments of humic acids or low molecular weight organic compounds (Fagnano et al. 2011), or from dissolved and colloid organic matter (Kaschl et al. 2002). Hence, the choice of the suitable kind of compost is a key point for the design of an efficient phytoremediation project. Anyway, the values here reported were of the same order of magnitude observed by Concas et al. (2015a) on a plot amended with compost of a previous field phytoremediation experiment (Bacchetta et al. 2012).

**Fig. 4:**
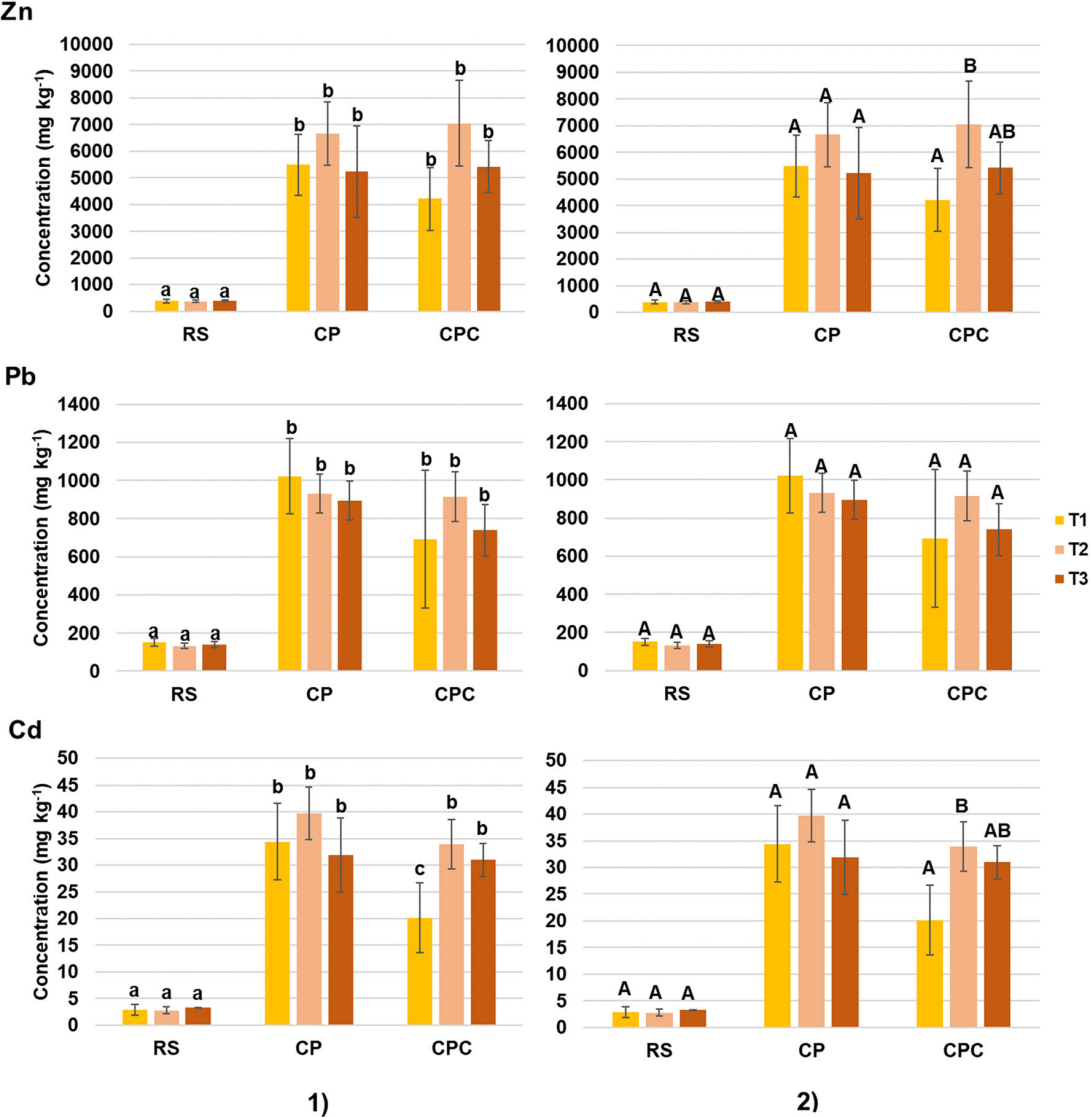
Concentration (mg kg^−1^) of Zn, Pb, and Cd (mean ± SD, n = 5) in the rhizosphere materials during the trial and related statistical analysis: **1** statistical analysis among treatments considered at a fixed time; **2** statistical analysis among time on the same matrix; different letters indicate statistically significant differences at p < 0.05; T1, after 1 month; T2, after 3 months; T3, after 6 months

The concentrations of Zn, Pb, and Cd in rhizosphere materials from T1 to T3 are reported in Fig. 4 and are consistent with those observed in the related substrates in terms of metal concentration, order of abundance of metals (Zn > Pb > Cd), and involved matrix (CP > CPC > RS). A fairly high heterogeneity was recognized also in the rhizosphere materials of CP and CPC, as already observed in the related substrates. Statistical analysis showed that metal concentration in rhizosphere was significantly different (p < 0.05) between contaminated matrices (CP and CPC) and unpolluted one (RS) for all metals during the experiment (Fig. 4, part 1). On the contrary, metal concentration into rhizosphere of CP and CPC was statistically similar for each metal tested (except for Cd at T1) showing that the addition of compost in mine waste had no effects in terms of reduction of Zn, Pb, and Cd concentration. Moreover, the concentrations of the three metal remained similar over time in each treatment (except for Zn and Cd in CPC; Fig. 4, part 2).

### Soil pore waters analysis

**Fig. 5:**
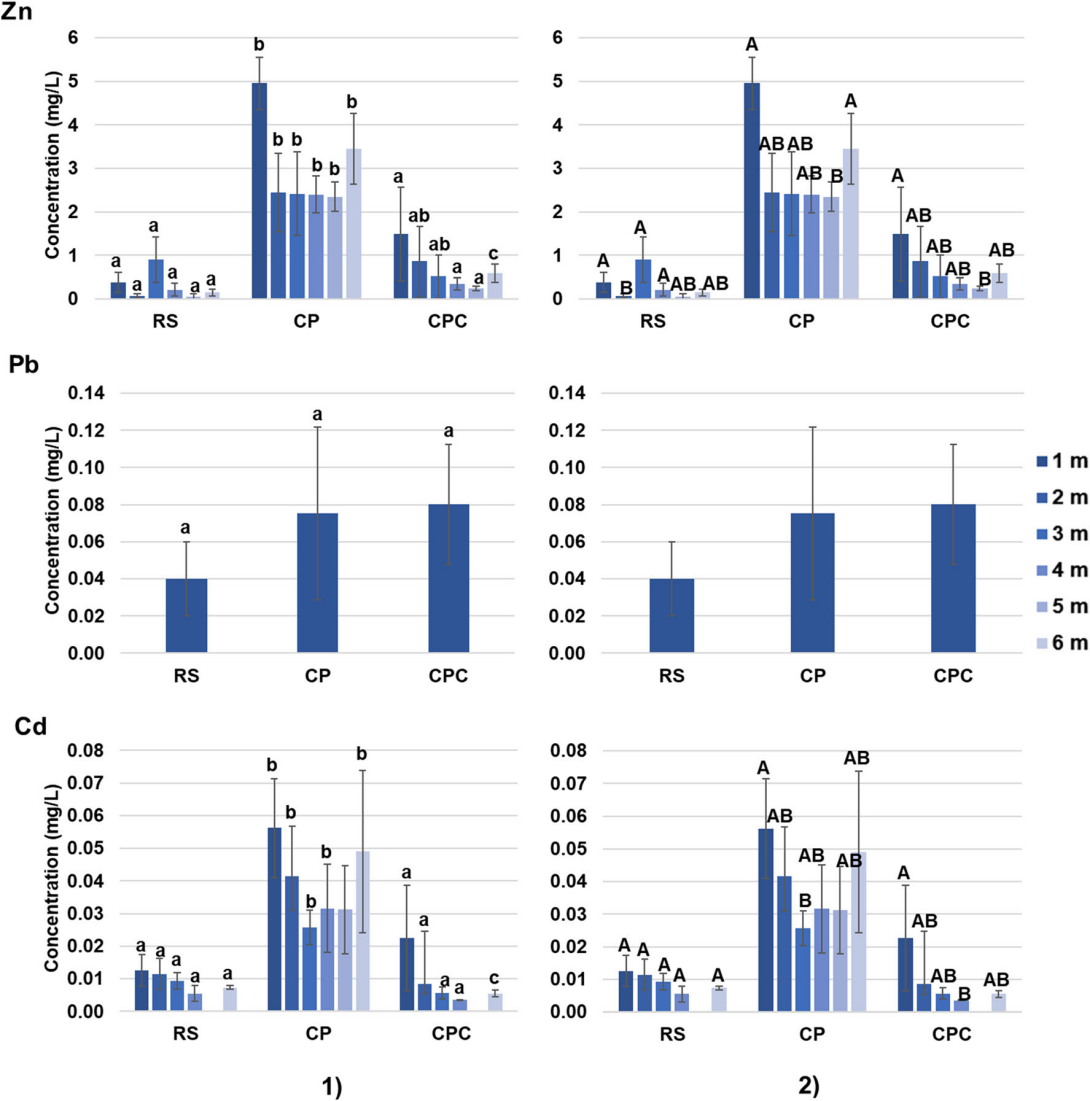
Zn, Pb, and Cd concentrations (mg/L) in soil pore waters during the experiment (mean ± SD; n = 5) and related statistical analysis: **1** statistical analysis of the metal content among treatments considered at a fixed time; **2** statistical analysis of the metal content at the different times (expressed in months); different letters indicate statistically significant differences at p < 0.05; 1 m, 2 m, 3 m, etc. indicate the number of months from the start of the experiment

The concentrations of Zn, Pb, and Cd in soil pore waters during all the experiment are shown in Fig. 5. Zn is the most abundant metal, followed by Pb and Cd. It is noteworthy that after the first month, the Pb concentrations were lower than the instrumental detection limit (< 0.02 mg/L). This fact may indicate that only a very smally quantity of Pb is soluble in water and it may have been depleted in the very first period of the experiment. Zinc and Cd were more concentrated in polluted matrices (CP > CPC), than in reference one (RS). The contents of Zn and Cd were significantly different (p < 0.05), with some exceptions, between contaminated matrices (CP and CPC; Fig. 5, part 1) and the addition of compost in the mine waste seems to be able to reduce the soluble fraction available to roots, decreasing the levels of these metals close the RS values. Some significant differences (p < 0.05) in terms of Zn and Cd concentrations were found in each matrix during the experiment (except for Cd in RS; Fig. 5, part 2). However, it is important to highlight that pore waters give information only about the soluble fraction but do not consider the solid constituents of soil, which take part of the total bioavailable pool (Adamo et al. 2013). The Zn and Cd values here reported are higher (approximately one order of magnitude) than those obtained by Concas et al. (2015b) in the same sampling site, whereas Pb concentrations were higher than those reported by the same authors but of the same order of magnitude. However, it is necessary to clarify that they sampled in field condition wherein the water availability is obviously uncontrollable if compared with a laboratory trial. When the metal availability is addressed, the soil pore waters’ sampling gives some advantages compared with indirect methods (i.e., single or sequential extractions) because it is more rapid and if it is used in field sampling can highlight differences in metals mobility during time (i.e., water seasonality).

### Mineralogical characterization of roots and metals accumulation in plant tissues

XRD analysis showed that roots were mainly composed of amorphous cellulose and quartz and no substantial differences were observed in their mineralogical composition among the different substrates. The formation of biominerals into plant tissue is a well-known phenomenon related to physiological needs and environmental stresses (He et al. 2014). The precipitation of mineral phases in plant tissues in presence of heavy metal pollution was observed in several plant species, like *Imperata cylindrica* (L.) Raeusch. (Rodríguez et al. 2005) and *Sarcocornia pruinosa* Fuente, Rufo & Sánchez Mata (De la Fuente et al. 2018) as reaction to environmental stress. Biominerals were detected also in Sardinian autochthonous plant species growing in extreme metal environments like *E. pithyusa* subsp. *cupanii* (Medas et al. 2015), *Juncus acutus* L. (Fancello et al. 2019; Medas et al. 2019), *Pistacia lentiscus* L. (De Giudici et al. 2015), and *Phragmites australis* (Cav.) Trin. ex Steud (De Giudici et al. 2017). A recent study (Boi et al. 2020a) has depicted the interaction among soil minerals and *H. tyrrhenicum* showing the presence of some biominerals in plant tissues (among them quartz, dolomite, and weddellite) likely as response to environmental stress. However, weddellite and dolomite were not detected in our work and more time would probably be needed to highlight an evolution in roots mineralogy.

**Fig. 6:**
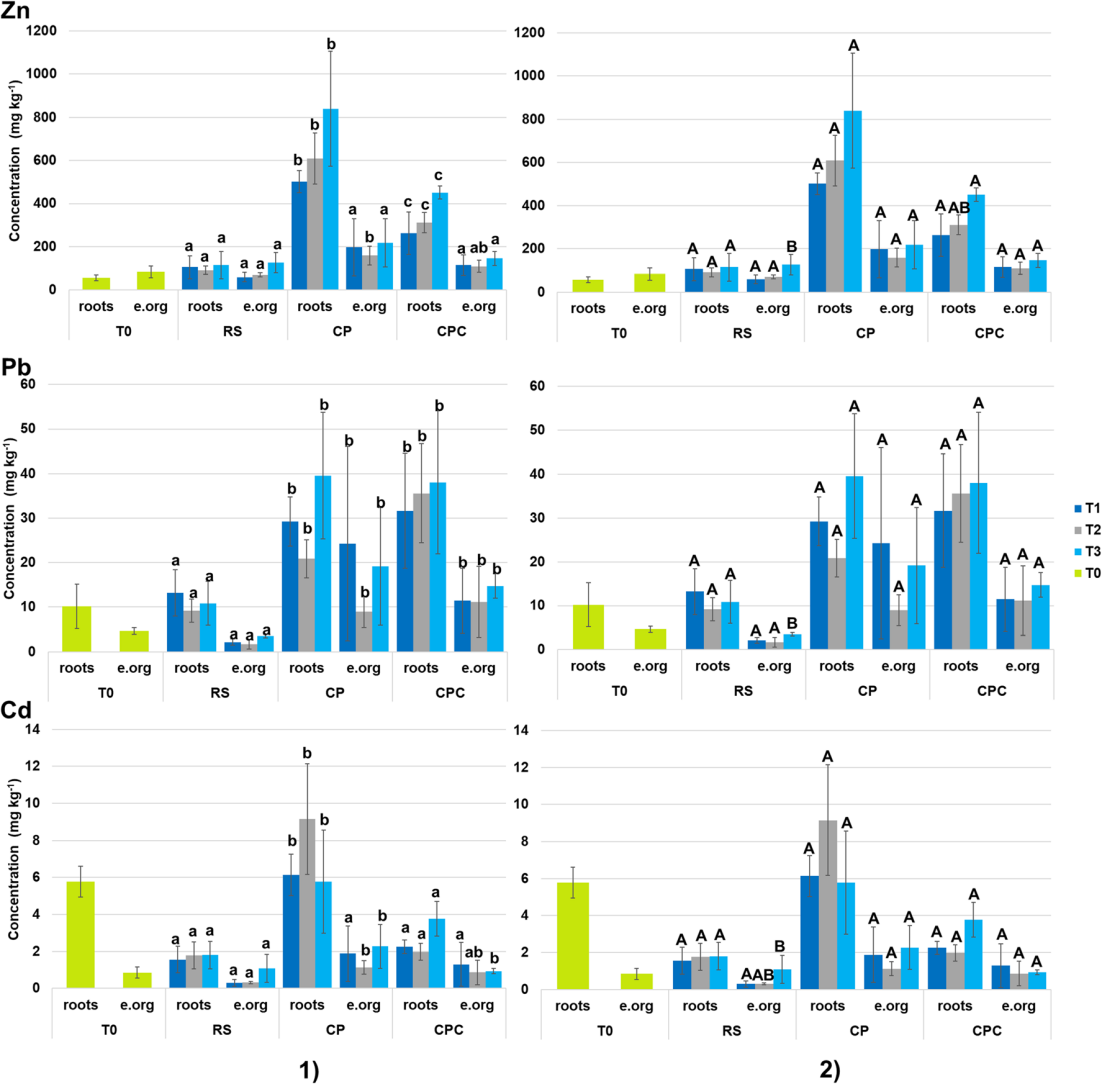
Zn, Pb, and Cd concentration (mg kg^−1^) in roots and epigean organs (e. org) of *H. tyrrhenicum* during the experiment (mean ± SD; n = 5) and related statistical analysis: **1** statistical analysis of the metal content among treatments considered at a fixed time; **2** statistical analysis of the metal content in each treatment over time (from T1 to T3); different letters indicate statistically significant differences at p < 0.05; T0, before planting; T1, after 1 month; T2, after 3 months; T3, after 6 months

The preliminary characterization of plant metal uptake at T0 (Table 3) showed that roots and epigean organs mainly accumulated Zn, followed by Pb and Cd, following the same order also after planting and throughout the phytoremediation test (from T0 to T3; Fig. 6). The metal concentration assessed at T0 (Table 3) was lower than those observed in plant tissues after the planting in the contaminated substrates (CP and CPC; Fig. 6). Furthermore, the three metals were generally more abundant in roots than in epigean organs. As far as root accumulation is concerned, the highest concentrations of Zn, Pb, and Cd were assessed in CP followed by CPC and RS. Statistical analysis highlighted, in some cases (see Fig. 6, part 1), significant differences (p < 0.05) between the three substrates in terms of roots uptake. In details, the accumulation of Zn was different in all substrates, and Pb was different between contaminated matrices (CP and CPC) and RS (Fig. 6, part 1), while regarding Cd accumulation in CP was different from both RS and CPC. Based on the findings of this study, compost may act differently depending on the involved metal (see Fig. 4). It is a well-known fact that organic amendments can reduce the metal uptake operated by roots, as reported for plant species growing in the same kind of polluted substrates of this study (Bacchetta et al. 2012, 2015). Despite the initial increase in terms of root’s metal content in the first month (from T0 to T1) of the trial, no significant differences (p > 0.05) were observed until the end of the trial (from T1 to T3) for each metal (Fig. 6, part 2). This fact could be reasonably due to the adaption of plant specimens to the new substrate conditions.

**Table 3:** Zn, Pb, and Cd concentration (mg kg^−1^) in plant tissues at T0 (mean ± SD n = 5)

	Zn (mg kg^−1^)	Pb (mg kg^−1^)	Cd (mg kg^−1^)
Roots	57.18 ± 13.41	10.20 ± 4.98	5.78 ± 0.83
Epigean organs	83.77 ± 25.54	4.62 ± 0.74	0.85 ± 0.31

Taking into account the accumulation of metals in epigean organs (Fig. 6, part 1), no differences were generally observed among substrates (p > 0.05) for Zn and Cd (with the exception of T2 for each metal), whereas the accumulation of Pb was significantly higher (p < 0.05) in CP and CPC, if compared with RS. Though compost can influence the metal uptake by roots, it does not appear to modify the accumulation of metals into epigean organs. Moreover, the accumulation of the three metals did not vary along time (p > 0.05) in CP and CPC; on the contrary, the metals uptake assessed in RS varied during the experiment in epigean organs (Fig. 6, part 2).

It is noteworthy that heavy metals are generally toxic for plants and living organisms even if some of them are essential for metabolism at low concentrations (i.e., Cu, Ni, and Zn; Nagajyoti et al. 2010). In particular, Zn is an essential nutrient for all plants and *H. tyrrhenicum* can catch a certain amount as micronutrient softening the remaining through a tolerance system, whereas Pb and Cd who are toxic and hardly accumulated by plants (Kabata-Pendias 2011) are well accumulated in *H. tyrrhenicum* tissues. Moreover, a recent study has shown that Zn is mainly present in the epidermis of roots of *H. tyrrhenicum*, indicating an exclusion system for this metal (Boi et al. 2020a). It is noteworthy that metal-tolerance capability is common of other plant species growing in the same area, like *Cistus salviifolius* L. (Jiménez et al. 2005, 2011, 2021), *P. lentiscus*, and *S. canina* subsp. *bicolor* (Bacchetta et al. 2012, 2015; Lai et al. 2015; Concas et al. 2015a). Similar behavior was observed also on other species of the genus *Helichrysum*, like *H. italicum* (Roth) G.Don subsp. *italicum* (Brunetti et al. 2017).

### Phytoremediation potential

Biological indexes were calculated in order to evaluate the phytoremediation potential of *H. tyrrhenicum*. As shown in Table S1 (see Online Resource), the BCFs were calculated using both the total and the bioavailable fraction (BCF *bf*) of the three metals. BCFs were < 1, with statistically significant differences (p < 0.05) among substrates. In details, BCFs assessed for Zn and Cd were different between the three substrates (with some exception, at T1 for Zn and at T3 for Cd, where CP and CPC did not show differences), whereas for Pb, the BCF was different between RS and the contaminated substrates (CP and CPC). The highest BCF was assessed for Zn and Cd in the unpolluted substrate and the lowest values assessed in CP and CPC may be due to plants ability to limit the uptake of toxic metals. Significant differences among sampling times (p < 0.05) were observed only in CP and CPC for Zn and Cd, whereas the uptake of Pb did not change (p > 0.05) in each substrate over time. The assessed BCF values showed that *H. tyrrhenicum* may have a different behavior in terms of roots uptake on the basis of the involved metals and substrates, indeed Zn and Cd were differently uptaken with evident differences among the three substrates and a clear effect operated by compost addition. Taking into account the bioavailable fraction, BCF *bf* was > 1 for every studied metal and, as observed for BCF, the behavior of the plant species towards metals significantly changed (p < 0.05) according to the involved substrate. In particular, *H. tyrrhenicum* behaved differently in CP and CPC, for each studied metal, showing again the effect operated by compost addition. Moreover, no significant differences among sampling times (p > 0.05) were highlighted throughout the trial for every substrate, except for Zn in CPC. The assessed BCF and BCF *bf* are consistent with those assesed in previous studies on this taxon (Cao et al. 2004; Bacchetta et al. 2017, 2018). Moreover, the BCF values recorded in this study were similar than those measured in *Dittrichia viscosa* (L.) Greuter subsp. *viscosa* (Jiménez et al. 2021), showing a similar root’s accumulation capability between this Asteraceae member.

BAC values reported in Table S2 (see Online Resource) were always < 1, with the highest values for Zn and Cd, especially in RS. Statistically significant differences among treatments (p < 0.05) were highlighted for Zn and Cd wherein the uptake in the areal organs was different between RS and the contaminated substrates (CP/CPC); on the contrary, no statistically significant differences (p > 0.05) between treatments were observed concerning the Pb’s BAC (except for T3). Significant differences among sampling times were observed (p < 0.05) only in the unpolluted substrate for all metals. As far as the bioavailable fraction is concerned, BAC *bf* was generally > 1 for each metal, and in particular, Zn showed the highest values, followed by Cd and Pb: RS showed the highest values for each metal and it is generally followed by CP and CPC. Statistical analysis highlighted significant differences (p < 0.05) among treatments for Zn and Cd, while no differences (p > 0.05) were observed for Pb (with the exception at T3). In particular, the uptake of bioavailable Zn and Cd in epigean organs was different between polluted matrices (CP/CPC) and RS. Moreover, significant differences among sampling times (p < 0.05) were generally observed in RS for Zn and Pb, whereas none (p > 0.05) was observed in CP and CPC for each metal. Hence, *H. tyrrhenicum* is able to uptake only a little concentration of metals into epigean organs as pointed out by BAC and BAC *bf* and the implementation of compost did not influence the uptake. Moreover, it is evident that the uptake into epigean organs did not vary in CP and CPC during all the trial, highlighting that *H. tyrrhenicum* catch only a certain amount of metals and showing again the potential presence of such a protective mechanism. The assessed BAC values (< 1) are consistent with other species that grow on polluted substrates as *D. viscosa* subsp. *viscosa*, *C. salviifolius*, and *E. pithyusa* subsp. *cupanii* (Jiménez et al. 2021).

The measured TF index, as reported in Table S3 (see Online Resource), was always < 1. The highest values were observed for Pb and Cd followed by Zn. No significant differences (p > 0.05) were observed among treatments for every studied metal (except for Zn in T2) as well as among sampling times (p > 0.05) in each substrate. Hence, when the translocation of metals from roots to epigean organs is considered, *H. tyrrhenicum* showed the same behavior in all substrates and during all the trial, showing also in this case the potential presence of a protective mechanism acting to limit metal translocation. Previous study carried out on this plant species (Cao et al. 2004; Bacchetta et al. 2017, 2018; Boi et al. 2020b) indicated TF values > 1. However, it can be noted that plants used in the studies of Bacchetta et al. (2017, 2018) were spontaneously grown in the Campo Pisano mine site, while in the study of Cao et al. (2004), a different substrate was used. As far as a comparison with other species is concerned, previous studies carried out on *C. salviifolius*, *S. canina* subsp. *bicolor*, and *D. viscosa* subsp. *viscosa* (Lai et al. 2015; Jiménez et al. 2021) showed TF higher than *H. tyrrhenicum*. Otherwise, in the study of Brunetti et al. (2017), *H. italicum* subsp. *italicum* have shown the capability to accumulate metals mainly into roots.

### Plant growth and survival

The highest plants survival percentage was recorded in RS (95%), followed by CPC (90%) and CP (80%), confirming again the great adaptability of *H. tyrrhenicum* to highly metal-polluted substrates, also without compost. Its addition to mine waste had a positive effect on survival, helping the specimens in their development. Comparing these survival data with the phytoremediation experiment carried out by Bacchetta et al. (2015), a higher survival percentage of *H. tyrrhenicum* in the same kind of matrix was recognized.

**Fig. 7:**
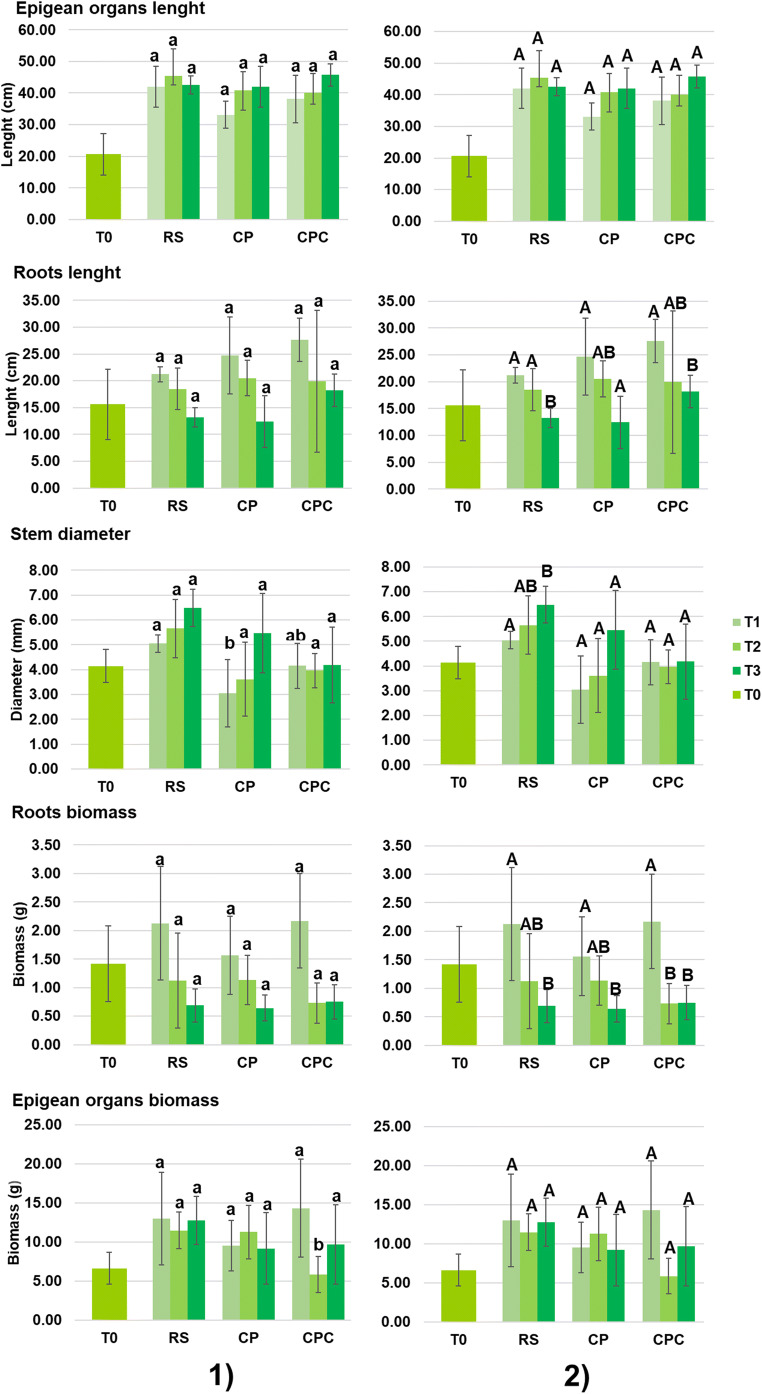
Biometric parameters of *H. tyrrhenicum* during the trial (mean ± SD; n = 5) and statistical analysis: **1** statistical analysis of biometric parameters among treatments considered at a fixed time; **2** statistical analysis of biometric parameters in each treatment over time (from T1 to T3); different letters indicate statistically significant differences at p < 0.05; T0, before planting; T1, after 1 month; T2, after 3 months; T3, after 6 months

As far as the growth of plant is concerned, Table 4 and Fig. 7 report the values of the biometric parameters measured on five specimens at the beginning of the phytoremediation experiment (T0) and their evolution assessed during the experiment, respectively. Statistical analysis showed no differences between treatments (p > 0.05) in terms of length of roots and epigean organs. In particular, roots length remained constant in the first month (from T0 to T1), but a significant length reduction (p < 0.05) was observed in each treatment (Fig. 7, part 2) during the rest of the experiment, whereas an elongation of epigean organs was generally observed from T0 to T1, but no statistically significant growth (p > 0.05) was observed from T1 to T3 in each substrate. Taking into account the diameter of the stem, no statistically significant differences (p > 0.05) were assessed between treatments (Fig. 7, part 1), except for T1 where the diameter was significantly higher in RS than CP/CPC. From T1 to T3, significant differences (p < 0.05) were observed in RS, corresponding with an enlargement, whereas in CP and CPC, the diameter remained constant (Fig. 7, part 2) during the experiment. Anyway, the diameter seems not to change if compared with measurement at T0 (Table 4). As for the roots and epigean biomass, no statistically significant differences (p > 0.05) between treatments were observed (with the exception at T2 for epigean organs biomass; Fig. 7, part 1). In details, the biomass of the roots did not change after 1 month from the beginning of the experiment (from T0 to T1), but a statistically significant decrease (p < 0.05) in weight was observed from T1 to T3 in all the treatments (Fig. 7). Conversely, the epigean organs biomass was constant (p > 0.05) throughout the experiment (p > 0.05) in all substrates. In addition, compost seems not to influence the growth of the roots and epigean organs, diameter of the stem, and biomass in *H. tyrrhenicum*. The metal pollution seems to operate mainly in terms of enlargement of stems; indeed, a significant enlargement was recorded only in RS, probably due to the lowest metal content: it is a well-known fact that metals can inhibit the water uptake (Kranner and Colville 2011) and as a consequence the enlargement of the stem.

**Table 4:** Biometric parameters of *H. tyrrhenicum* at the beginning of the phytoremediation experiment (T0; mean ± SD; n = 5)

Biometric parameters	
Epigean organs length	20.60 ± 6.58 cm
Roots length	15.60 ± 6.58 cm
Stem diameter	4.14 ± 0.36 mm
Roots biomass	4.14 ± 0.36 g
Epigean organs biomass	6.62 ± 2.05 g

## Conclusion

This ex situ phytoremediation experimental study evaluated the phytostabilization potential of *H. tyrrhenicum*, suggesting “*ex-situ* experiments” as an important source of informations when planning recovery actions. Results showed that *H. tyrrhenicum* accumulated high concentrations of Zn, Pb, and Cd into roots, mirroring the high contamination levels observed in the polluted substrates. The assessed values of these metals in the considered mine substrate are higher than the established threshold limits of Italian law for industrial use of soil and consequently, hindering the possibility of using these areas. A “gentle remediation,” as also proposed by Jiménez et al. (2021), can give an important contribution for both remediation and future use of these sites. Taking account of the assessed metal concentrations and BAC, BCF, and TF values, this study emphasizes the phytostabilization potential of *H. tyrrhenicum.* Moreover, the high plant survival and the assessed growth parameters confirmed the great adaptability of *H. tyrrhenicum* to the limiting ecological conditions of mine sites, also without amendments. Anyway, the addition of compost created the optimal compromise for plant establishment: on one side, it decreased the metal accumulation into roots; on the other one, it helped in the development of the specimens by increasing the survival. Owing to these reasons, compost can be suggested as an effective amendment in phytostabilization activities of these areas. Future investigation could concern an in situ phytoremediation experiment using seedlings grown naturally in the mine areas, with the aim to shed light about the survival and the metal uptake in this sensitive step of a plant life under field condition.

## Supplementary Information

ESM 1 (DOCX 33 kb)
