# Supplementary material for: Ex situ phytoremediation trial of Sardinian mine waste using a pioneer plant species
Source: Environ Sci Pollut Res Int. 2021 Jun 18;28(39):55736–53. doi: 10.1007/s11356-021-14710-y (PMC8494672; doi:10.1007/s11356-021-14710-y)
Supplement: Supplementary file 1 — (DOCX 33 kb) [file 11356_2021_14710_MOESM1_ESM.docx]

**ONLINE SUPPLEMENTARY MATERIALS**

**Title:**

***Ex situ* phytoremediation trial of Sardinian mine waste using a pioneer plant species**

**Journal:**

[Environmental Science and Pollution Research](https://www.springer.com/journal/11356/)

**Authors:**

Maria Enrica Boi^1^, Giovanna Cappai^2^, Giovanni De Giudici^1^, Daniela Medas^1^, Martina Piredda^2^, Marco Porceddu^3,4*^, Gianluigi Bacchetta^3,4^

^1^ Department of Chemical and Geological Sciences, University of Cagliari, Cittadella Universitaria di Monserrato, S.S. 554 bivio per Sestu, 09042 Monserrato (CA), Italy

^2^ Department of Civil and Environmental Engineering and Architecture, University of Cagliari, Piazza d’Armi 1, 09123 Cagliari, Italy

^3^ Department of Life and Environmental Sciences, Centre for Biodiversity Conservation (CCB), University of Cagliari, Viale Sant’Ignazio da Laconi 11-13, 09123 Cagliari, Italy

^4^ Sardinian Germplasm Bank (BG-SAR), Hortus Botanicus Karalitanus (HBK), University of Cagliari, Viale Sant’Ignazio da Laconi, 9-11, 09123, Cagliari, Italy

*Corresponding author: Marco Porceddu, [porceddu.marco@unica.it](mailto:porceddu.marco@unica.it); telephone number: +39-0706753806

**Table S1:** BCF and BCF*_bf_* of Zn, Pb and Cd in *H. tyrrhenicum* (mean ± SD; n = 5) and related statistical analysis among treatments (lower case letters) and for each treatment over time (upper case letters); different letters indicate statistically significant differences at p < 0.05; T1 = after one month; T2 = after three months; T3 = after six months.

|  |  |  | RS | CP | CPC |
| --- | --- | --- | --- | --- | --- |
| Zn | **BCF** | **T1** | 0.33 ± 0.16^a; A^ | 0.05 ± 0.01^b; A^ | 0.04 ± 0.02^b; A^ |
|  |  | **T2** | 0.39 ± 0.09^a; A^ | 0.07 ± 0.01^b; A^ | 0.04 ± 0.01^c; A^ |
|  |  | **T3** | 0.76 ± 0.42^a; A^ | 0.12 ± 0.04^b; B^ | 0.06 ± 0.01^c; B^ |
|  | **BCF*_bf_*** | **T1** | 17.04 ± 8.37^a; A^ | 10.00 ± 1.00^a; A^ | 1.74 ± 0.65^b; A^ |
|  |  | **T2** | 14.76 ± 3.19^a; A^ | 12.13 ± 2.36^a; A^ | 2.08 ± 0.31^b; AB^ |
|  |  | **T3** | 18.6 ± 10.28^a; A^ | 16.79 ± 5.29^a; A^ | 3.02 ± 0.2^b; B^ |
| Pb | **BCF** | **T1** | 0.10 ± 0.04^a; A^ | 0.02 ± 0.00^b; A^ | 0.03 ± 0.01^b; A^ |
|  |  | **T2** | 0.09 ± 0.03^a; A^ | 0.011 ± 0.002^b; A^ | 0.02 ± 0.01^c; A^ |
|  |  | **T3** | 0.13 ± 0.06^a: A^ | 0.02 ± 0.01^b; A^ | 0.03 ± 0.01^b; A^ |
|  | **BCF*_bf_*** | **T1** | 4.49 ± 1.77^a; A^ | 3.38 ± 0.64^a; A^ | 1.52 ± 0.61^b; A^ |
|  |  | **T2** | 3.12 ± 0.89^a; A^ | 2.42 ± 0.50^a; A^ | 1.70 ± 0.53^a; A^ |
|  |  | **T3** | 3.69 ± 1.66^ab; A^ | 4.58 ± 1.65^a; A^ | 1.82 ± 0.77^b; A^ |
| Cd | **BCF** | **T1** | 0.60 ± 0.28^a; A^ | 0.12 ± 0.02^b; A^ | 0.09 ± 0.01^c; A^ |
|  |  | **T2** | 0.91 ± 0.37^a; A^ | 0.23 ± 0.08^b; B^ | 0.05 ± 0.01^c; B^ |
|  |  | **T3** | 1.54 ± 0.64^a; A^ | 0.12 ± 0.06^b; AB^ | 0.07 ± 0.02^b; A^ |
|  | **BCF*_bf_*** | **T1** | 17.31 ± 8.01^a; A^ | 11.37 ± 2.08^a; A^ | 1.03 ± 0.16^b; A^ |
|  |  | **T2** | 19.74 ± 8.10^a; A^ | 16.96 ± 5.43^a; A^ | 0.91 ± 0.20^b; A^ |
|  |  | **T3** | 20.01 ± 8.29^a; A^ | 10.71 ± 5.16^a; A^ | 1.73 ± 0.43^b; A^ |

**Table S2:** BAC and BAC*_bf_* of Zn, Pb and Cd in *H. tyrrhenicum* (mean ± SD; n = 5) and related statistical analysis among treatments (lower case letters) and for each treatment over time (upper case letters); different letters indicate statistically significant differences at p < 0.05; T1 = after one month; T2 = after three months; T3 = after six months

|  |  |  | **RS** | **CP** | **CPC** |
| --- | --- | --- | --- | --- | --- |
| **Zn** | **BAC** | **T1** | 0.189 ± 0.064^a; A^ | 0.021 ± 0.014^b; A^ | 0.019 ± 0.008^b; A^ |
|  |  | **T2** | 0.305 ± 0.038^a; A^ | 0.020 ± 0.005^b; A^ | 0.014 ± 0.004^b; A^ |
|  |  | **T3** | 0.834 ± 0.310^a; B^ | 0.029 ± 0.020^b; B^ | 0.030 ± 0.019^b; B^ |
|  | **BAC*_bf_*** | **T1** | 9.73 ± 3.30^a; A^ | 3.93 ± 2.63^a; A^ | 0.77 ± 0.31^b; A^ |
|  |  | **T2** | 11.41 ± 1.44^a; A^ | 3.17 ± 0.88^b; A^ | 0.73 ± 0.18^c; A^ |
|  |  | **T3** | 20.53 ± 7.63^a; B^ | 4.07 ± 2.76^b; A^ | 1.58 ± 1.01^b; A^ |
| **Pb** | **BAC** | **T1** | 0.016 ± 0.005^a; A^ | 0.013 ± 0.012^a; A^ | 0.011 ± 0.007^a; A^ |
|  |  | **T2** | 0.017 ± 0.011^a; A^ | 0.005 ± 0.002^a; A^ | 0.007 ± 0.005^a; A^ |
|  |  | **T3** | 0.042 ± 0.004^a; A^ | 0.012 ± 0.008^b; A^ | 0.012 ± 0.002^b; A^ |
|  | **BAC*_bf_*** | **T1** | 0.74 ± 0.23^a; AB^ | 2.81 ± 2.52^a; A^ | 0.55 ± 0.35^a; A^ |
|  |  | **T2** | 0.58 ± 0.37^a; A^ | 1.04 ± 0.41^a; A^ | 0.53 ± 0.38^a; A^ |
|  |  | **T3** | 1.19 ± 0.12^a; B^ | 2.22 ± 1.53^a; A^ | 0.71 ± 0.13^a; A^ |
| **Cd** | **BAC** | **T1** | 0.114 ± 0.066^a; A^ | 0.038 ± 0.030^a; A^ | 0.050 ± 0.046^a; A^ |
|  |  | **T2** | 0.159 ± 0.028^a; A^ | 0.028 ±0.009^b; A^ | 0.024 ± 0.018^b; A^ |
|  |  | **T3** | 0.929 ± 0.643^a; A^ | 0.047 ± 0.025^b; A^ | 0.018 ± 0.003^c; A^ |
|  | **BAC*_bf_*** | **T1** | 3.29 ± 1.90^a; A^ | 3.49 ± 2.78^a; A^ | 0.60 ± 0.55^a; A^ |
|  |  | **T2** | 3.44 ± 0.60^a; A^ | 2.09 ± 0.69^b; A^ | 0.40 ± 0.31^c; A^ |
|  |  | **T3** | 12.07 ± 8.35^a; A^ | 4.21± 2.21^a; A^ | 0.43 ± 0.06^b; A^ |

**Table S3:** TF of Zn, Pb and Cd in *H. tyrrhenicum* (mean ± SD; n = 5) and related statistical analysis among treatments (lower case letters) and for each treatment over time (upper case letters); different letters indicate statistically significant differences at p < 0.05; T1 = after one month; T2 = after three months; T3 = after six months.

|  |  | **RS** | **CP** | **CPC** |
| --- | --- | --- | --- | --- |
| **TF Zn** | **T1** | 0.69 ± 0.42^a; A^ | 0.41 ± 0.31^a; A^ | 0.46 ± 0.13^a; A^ |
|  | **T2** | 0.79 ± 0.15^a; A^ | 0.28 ± 0.13^b; A^ | 0.36 ± 0.08^b; A^ |
|  | **T3** | 1.28 ± 0.72^a; A^ | 0.30 ± 0.32^a; A^ | 0.31 ±0.06^a; A^ |
| **TF Pb** | **T1** | 0.19 ± 0.08^a; A^ | 0.82 ± 0.69 ^a; A^ | 0.37 ± 0.17^a; A^ |
|  | **T2** | 0.19 ± 0.13^a; A^ | 0.44 ± 0.18^a; A^ | 0.34 ± 0.26^a; A^ |
|  | **T3** | 0.33 ± 0.11^a; A^ | 0.58 ± 0.42^a; A^ | 0.59 ± 0.53^a; A^ |
| **TF Cd** | **T1** | 0.20 ± 0.08^a; A^ | 0.32 ± 0.27^a; A^ | 0.65 ± 0.73^a; A^ |
|  | **T2** | 0.19 ± 0.05^a; A^ | 0.14 ± 0.07^a; A^ | 0.43 ± 0.26^a; A^ |
|  | **T3** | 0.62 ± 0.47^a; A^ | 0.51 ± 0.35^a; A^ | 0.28 ± 0.14^a; A^ |
